# A Role for the CAL1-Partner Modulo in Centromere Integrity and Accurate Chromosome Segregation in *Drosophila*

**DOI:** 10.1371/journal.pone.0045094

**Published:** 2012-09-21

**Authors:** Chin-Chi Chen, Elizabeth Greene, Sarion R. Bowers, Barbara G. Mellone

**Affiliations:** Department of Molecular and Cell Biology, University of Connecticut, Storrs, Connecticut, United States of America; University of Virginia, United States of America

## Abstract

The relationship between the nucleolus and the centromere, although documented, remains one of the most elusive aspects of centromere assembly and maintenance. Here we identify the nucleolar protein, Modulo, in complex with CAL1, a factor essential for the centromeric deposition of the centromere-specific histone H3 variant, CID, in *Drosophila*. Notably, CAL1 localizes to both centromeres and the nucleolus. Depletion of Modulo, by RNAi, results in defective recruitment of newly-synthesized CAL1 at the centromere. Furthermore, depletion of Modulo negatively affects levels of CID at the centromere and results in chromosome missegregation. Interestingly, examination of Modulo localization during mitosis reveals it localizes to the chromosome periphery but not the centromere. Combined, the data suggest that rather than a direct regulatory role at the centromere, it is the nucleolar function of modulo which is regulating the assembly of the centromere by directing the localization of CAL1. We propose that a functional link between the nucleolus and centromere assembly exists in *Drosophila*, which is regulated by Modulo.

## Introduction

The centromere is a distinct region of the chromosome, which is required and necessary for accurate chromosome segregation during cell division because it provides the physical platform for kinetochore formation and spindle attachment. Interestingly, centromere formation is not dependent on the primary DNA sequence, but is instead defined epigenetically. Specifically, centromeric chromatin is marked by the presence of the histone H3-variant CENP-A (CID in *Drosophila*) [1], [2]. CENP-A deposition is mediated by the CENP-A-specific chaperone HJURP in vertebrates [3], [4], and by the related Scm3 in yeast [5]–[9]. However, no homolog of HJURP or Scm3 exists in *Drosophila*. Instead, Chromosome Alignment 1, CAL1, has been shown to share some degree of functional homology with HJURP and Scm3 and is essential for CID centromeric localization [10]–[12]. Like HJURP and CENP-A in humans, CAL1 and CID interact in prenucleosomal complexes, and CAL1 is recruited to the centromere at approximately the same time as when CID is deposited [12]. The precise role of CAL1 is currently unknown, although it has been proposed that it may function by ‘bridging’ together CID and CENP-C [13].

One aspect of centromere assembly that remains particularly elusive is the physical and functional relationship with the nucleolus. Centromeres cluster around nucleoli during various stages of the cell cycle [14]–[17] although what role this plays in centromere assembly is unclear. One suggestion is that the nucleolus sequesters a number of the factors required for centromere assembly and regulation and releases these proteins at specific cell cycle stages, providing a mechanism whereby these factors can be assembled at the centromere at specific times, thus allowing parts of the centromere and the kinetochore structure to be assembled transiently [17]. By clustering to the nucleolus, the centromere is in spatial proximity, allowing this regulatory mechanism to take place. Alongside clustering to the nucleolus, a number of centromeric and kinetochore factors have been found localized to the nucleolus. In humans the conserved centromere protein CENP-C and inner-centromere protein INCENP are enriched at the nucleolus during interphase [17]. Moreover, human CENP-C contains a nucleolar localization sequence (NoLS) that is essential for its function [17] and interacts with two related nucleolar transcription factors, UBF and NOR90 [18]. Interestingly, UBF also indirectly interacts with another centromere protein, CENP-F, via the retinoblastoma protein, thereby providing another association between a centromere protein and a nucleolar factor [19]. Perhaps most interesting of all is the discovery that the human CENP-A chaperone, HJURP, also localizes to the nucleolus during interphase [20], [21]. Notably, the association of HJURP with the nucleolus is cell-cycle dependent, with HJURP being detected at the nucleolus in increasing levels throughout S-phase. HJURP then localizes to the centromere during telophase/early G1, precisely at the same time when CENP-A is loaded [22]. Along with its centromeric localization, CAL1 also localizes to the nucleolus in interphase [10], and this localization is mediated by the middle part of CAL1 (residues 392–722), a highly variable region that is dispensable for centromeric localization [13].

In addition to centromeric proteins associating at the nucleolus, nucleolar proteins have been found associated with centromeric factors. Nucleophosmin, a nucleolar protein, has been found specifically associated with nucleosomes containing CENP-A but not H3 [23]. Nucleophosmin (NPM1) has been found associated with CENP-A-H4-HJURP prenucleosomal complexes [3], [23], however it is unclear whether NPM1 is required for CENP-A deposition [3]. It has been suggested that NPM1, which is known to act as a chaperone for H3-H4 and H2A-H2B [24], [25] and is associated with ATP [26], might have ATPase activity which would allow it to facilitate the chromatin remodeling/assembly activity that occurs during CENP-A deposition [3]. An alternative suggestion is that NPM1 plays a role in kinetochore assembly as it has been found with CENP-W [27], an essential partner of CENP-T [23] a centromere protein essential for kinetochore assembly [28]. In support of the idea that NPM1 is required for centromere and/or kinetochore assembly, depletion of NPM1 in human HeLa cells has been shown to cause chromosome missegregation as well as a number of other defects [27], [29].

To gain insight into the function of CAL1 in the CID assembly pathway, we carried out a mass-spectrometry screen of CAL1 complexes obtained by FLAG-CAL1 immunoprecipitation (S. Bowers and B. Mellone, unpublished). One of the factors identified was Modulo, a factor which has been shown to localize to the nucleolus in *Drosophila* embryos [30] and is specifically required for growth of proliferative cells as a result of its association with the proto-oncogene Myc [31]. Modulo is structurally related to the nucleolar protein Nucleolin, a regulator of chromatin structure [32]. Nucleolin homologs are found in many species, are characterized by their ability to bind both RNA and DNA [32], and are associated with rDNA transcription [33] and rRNA maturation [34]. In line with this, Modulo is able to bind DNA and RNA. Interestingly, the DNA binding domain of Modulo is sequence-specific while the RNA binding domain is not [35]. Modulo has been suggested to be involved in a number of functions and early studies found it was essential for transcription of spermatid-differentiation genes and supported high expression of meiotic arrest genes [36]. In addition, in common with Nucleolin, Modulo is phosphorylated, and it is this phosphorylation that serves to regulate Modulo localization. Nucleolar Modulo is phosphorylated while the chromatin-associated Modulo is not [35]. As centromeric RNAs have also been found associated with the nucleolus [17], this raises the possibility that Modulo binds these centromeric RNAs providing another level of centromeric regulation. It is important to note that CAL1, like HJURP, localizes to the nucleolus as well as to the centromere [10]. However, it is unclear whether this localization is functionally relevant given the observation that fly CAL1 mutants lacking the region responsible for CAL1's nucleolar localization are viable [13].

Here, we investigate the role of Modulo in centromere function. We find that Modulo regulates the nucleolar localization of CAL1, and that loss of Modulo results in decreased levels of CID at the centromere and results in chromosome missegregation. We discuss possible mechanisms to account for the role of Modulo in centromere function.

## Results

### Isolation of the nucleolar protein Modulo from CAL1 immunoprecipitates

**Figure 1:**
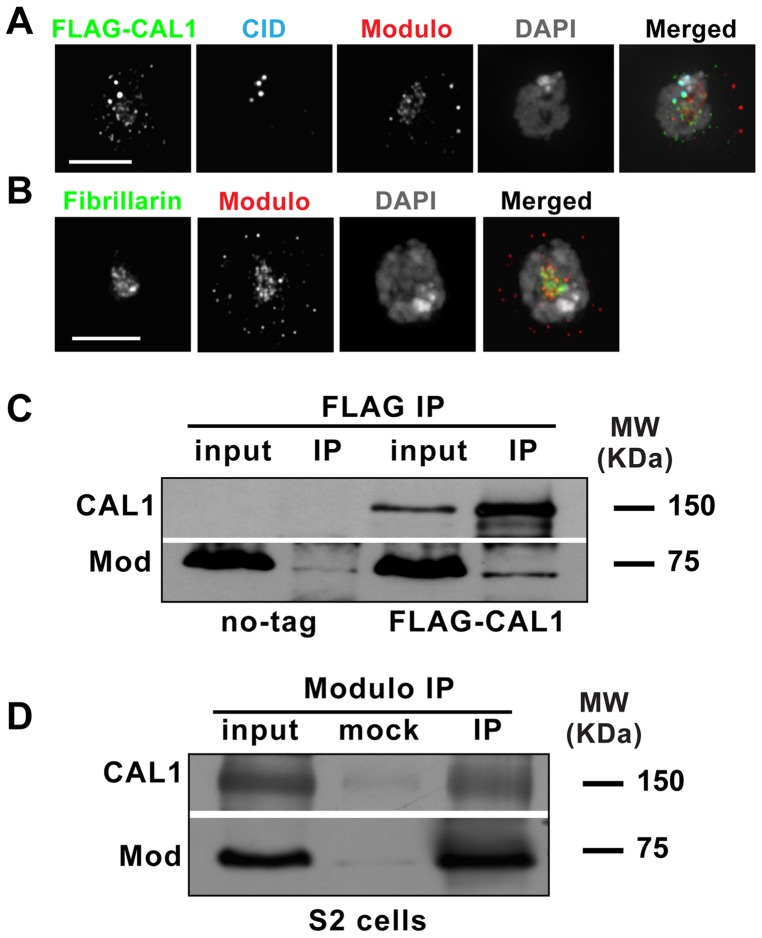
Identification of the CAL1 partner, Modulo. A) Immunofluorescence of S2 cell stably expressing FLAG-CAL1 showing colocalization between FLAG-CAL1 and CID. FLAG is shown in green, CID in blue, Modulo in red and DAPI in gray. Bar 5 µm. B) Immunofluorescence of S2 cell showing co-localization of Modulo (red) and Fibrillarin (nucleolar marker, green). DAPI is shown in gray. Bar 5 µm. C) Western blots of IPs carried out with anti-FLAG beads in untransfected S2 cells (no tag) and cells stably expressing FLAG-CAL1. The top Western blot shows the absence of CAL1 in the no-tag and its presence in the FLAG-CAL1 input and IP. The bottom Western blot shows the presence of Modulo in the input of both no-tag and FLAG-CAL1 and the enrichment of Modulo in the FLAG-CAL1 IP. Modulo runs as a 75 KDa protein while CAL1 runs approximately as a 150 KDa protein. D) Western blots of IPs carried out with beads coupled to anti-Modulo antibodies. Mock indicates control IP where the addition of anti-Modulo antibody was omitted. CAL1 is visible in IPs with the antibody and not in the mock IP.

In an effort to elucidate the role of CAL1 in centromere function, we carried out large-scale purifications using *Drosophila* S2 cells stably expressing a FLAG-CAL1 N-terminus fusion expressed under the endogenous CAL1 promoter. In this stable line, FLAG-CAL1 localized to centromeres and the nucleolus, consistent with previous reports (Fig. 1A). We focused on the identification of CAL1-partners from pre-nucleosomal complexes, with the goal of identifying novel regulators of centromere assembly. Chromatin-free extracts were generated as described [12] from FLAG-CAL1 and untagged S2 cells and immunoprecipitations (IP) using FLAG-beads were carried out. After extensive washes, bound complexes were eluted and submitted for LC-MS/MS analysis. This analysis yielded many putative CAL1 partners, which will be described and characterized elsewhere, and included the nucleolar protein Modulo [37]. Immunofluorescence (IF) shows that Modulo and CAL1 partially overlap at the nucleolus (identified by the presence of the nucleolar marker Fibrillarin) (Fig. 1A–B). To confirm whether Modulo is a CAL1 partner, we carried out IPs from total nuclear extracts from FLAG-CAL1 expressing cells and untagged S2 cells using anti-FLAG beads and performed Western blot analysis with specific anti-CAL1 and anti-Modulo antibodies [10], [38]. Quantification of the Modulo signal in the IP from FLAG-CAL1 cells compared to that from untagged S2 cells showed a five fold enrichment of Modulo in the FLAG-CAL1 IPs (Fig. 1C), confirming the specificity of the interaction between CAL1 and Modulo. In these IPs we also detected enrichment of FLAG-CAL1 as expected (Fig. 1C). We also carried out reciprocal IPs from total nuclear extracts obtained from S2 cells, using anti-Modulo antibody bound to beads. Western blot analysis detected Modulo itself (Fig. 1D) and CAL1 (enriched eight fold relative to the mock IP), further confirming their interaction.

### Modulo does not localize to centromeres

**Figure 2:**
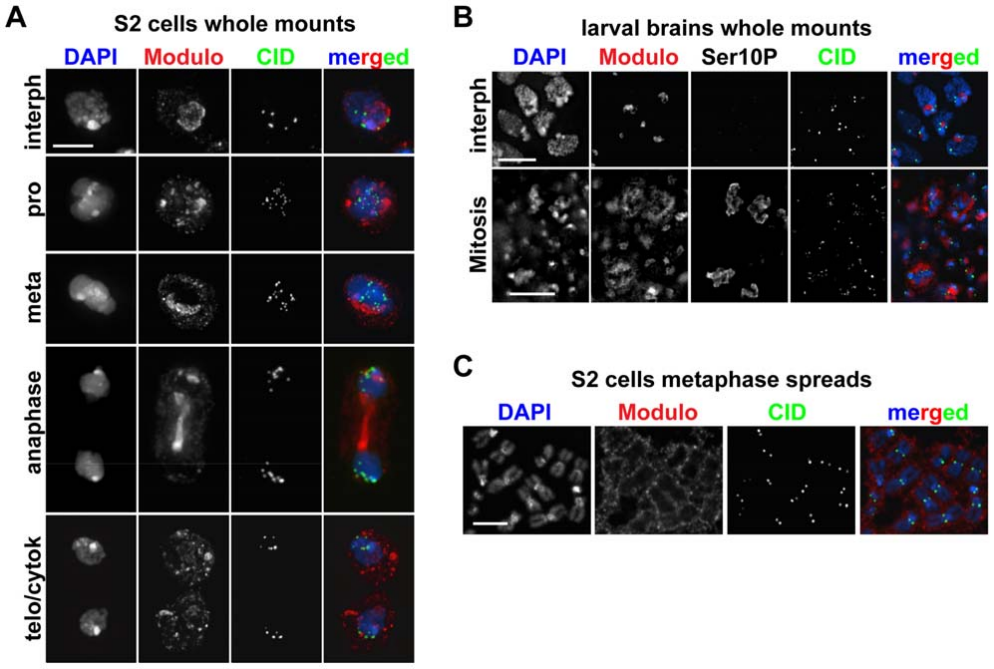
Analysis of Modulo localization in S2 cells and the larval brain. A) IF with anti-Modulo antibody (red) in S2 cells in interphase and at different mitotic stages (as indicated). Co-staining with anti-CID antibodies (green), shows the lack of significant overlap between the two proteins. DAPI is shown in blue. Bar 5 µm. B) IF with anti-Modulo antibody (red) in larval brain squashes. H3 Ser10p staining indicates mitotic cells. Co-staining with CID (green) confirms the lack of co-localization with Modulo. Bar 20 µm. C) IF with anti-Modulo antibody (red) and anti-CID (green) on mitotic chromosome spreads from S2 cells. DAPI is shown in blue. Bar 1 µm.

Previous studies established that Modulo broadly localizes to chromatin as well as to the nucleolus in *Drosophila* embryos [30]. Given our observation that Modulo interacts with CAL1 in S2 cells, we wanted to analyze, for the first time, the localization of Modulo at higher resolution and at different cell cycle stages and to determine whether or not Modulo also localizes to centromeres. Immunofluorescence (IF) was performed in S2 cells to detect Modulo and the centromere-marker CID using specific antibodies. In interphase, we confirmed that Modulo accumulates at the nucleolus and has a weaker staining on DNA (visualized by DAPI staining), however, we did not observe any co-localization with the CID signal (Fig. 2A, first row). During prophase in S2 cells, Modulo accumulated in clusters that did not overlap with DNA, which likely reflected the nucleolar fraction of Modulo being disassembled with the rest of the nucleolus at this stage (Fig. 2A, second row). In mitosis, Modulo localized in diffused speckles that persisted through cytokinesis until the formation of nucleoli in the next interphase (Fig. 2A, rows 3–5). These observations were also confirmed in cells in interphase and mitosis from larval brain squashes (Fig. 2B). To visualize the metaphase localization in more detail, we performed IF on metaphase spreads from S2 cells and confirmed the diffused localization of Modulo and the lack of co-localization with centromeres. Metaphase spreads showed localization of Modulo around the periphery of chromosomes (Fig. 2C), consistent with observations for other nucleolar proteins [39]. Based on these observations, we propose that Modulo and CAL1 physically interact primarily within the nucleolus rather than at the centromere. However, it is possible that Modulo is present at the centromeres at levels too low to be detectable under our experimental conditions.

### Knock-down of Modulo by RNAi causes loss of nucleolar CAL1

**Figure 3:**
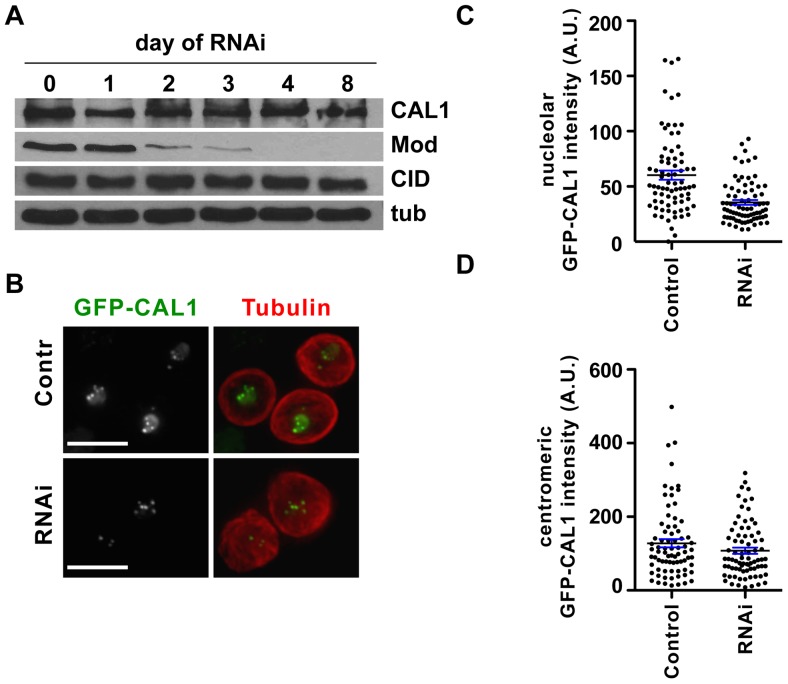
Modulo RNAi causes mislocalization of GFP-CAL1. A)Western blot analysis of total extracts from a Modulo RNAi time-course experiment. On day 0 cells were treated with dsRNA. An identical number of cells was taken every 24 h for 8 days and cell extracts were loaded on and SDS-PAGE. Western blots with anti-CAL1 and anti-CID antibodies show no visible changes in CAL1 and CID levels while Modulo became undetectable by day 4. Tubulin is shown as loading control. B) Images of control and Modulo RNAi (RNAi) live cells expressing GFP-CAL1 (green) and mCherry-tubulin (red), shown here as a counterstaining. Note the decrease in the nucleolar GFP-CAL1 upon Modulo RNAi. Bar 10 µm. C) Quantification of the nucleolar GFP-CAL1 signal by scatter dot plot. Dots represent the total GFP-CAL1 signal for individual cells. Black line: average signal, blue error bars: standard error. D) Scatter dot plot of the centromeric GFP-CAL1 signal in control untransfected cells versus Modulo RNAi cells (RNAi). Black line: average signal, blue error bars: standard error.

Having established that Modulo is a CAL1 partner, we next investigated whether it plays a role in CAL1 function. To address this, RNAi knock-down of Modulo was performed in S2 cells expressing GFP-CAL1 and mCherry-tubulin [10]. Western blot analysis determined that Modulo protein levels decreased to undetectable levels 4 days after addition of double stranded RNA (dsRNA), whereas levels of CAL1 and CID were unaffected (Fig. 3A). We analyzed the localization of GFP-CAL1 in live cells that also expressed mCherry-tubulin under these conditions. In control cells, GFP-CAL1 localized to centromeres and to the nucleolus as reported previously (Fig. 3B and [10]). Cells that underwent Modulo RNAi showed normal centromeric levels of GFP-CAL1, however, the nucleolar signal appeared significantly reduced (Fig. 3B). Quantification of the GFP-CAL1 signal confirmed that nucleolar GFP-CAL1 was significantly reduced in RNAi treated cells compared to control cells (42% reduction; n = 76 and n = 77, respectively; p<0.0001, unpaired t-test; Fig. 3C), while the centromeric GFP-CAL1 signal appeared unaffected (p = 0.15, unpaired t-test; Fig. 3D). Since Western blot analysis showed that total CAL1 protein did not decrease upon Modulo RNAi (Fig. 3A), it is possible that the nucleolar GFP-CAL1 lost upon Modulo RNAi becomes broadly distributed throughout the nucleus and is not degraded. We conclude that Modulo is required for the correct localization of GFP-CAL1 at the nucleolus, while it is dispensable for GFP-CAL1 localization at the centromere.

### Modulo is required for the recruitment of newly synthesized CAL1 at centromeres

**Figure 4:**
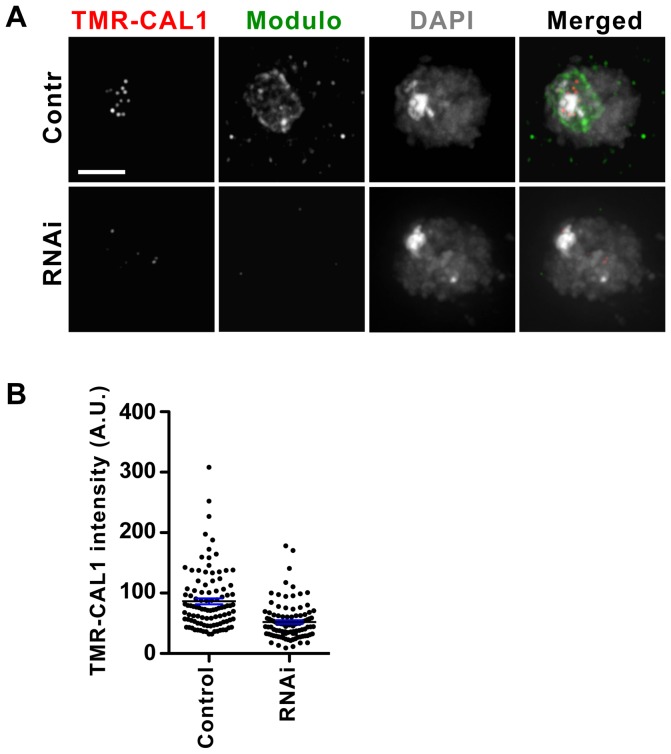
Modulo is required for newly synthesized CAL1 centromere recruitment. A) S2 cells stably expressing SNAP-CAL1 were subjected to Modulo RNAi (RNAi) or mock treated (control). A quench-chase-pulse experiment detected the newly synthesized SNAP-CAL1 pool (TMR-CAL1, red). Staining with anti-Modulo (green) by IF confirmed Modulo depletion in RNAi cells. DAPI is shown in gray. Bar 5 µm. B) Quantification of the total centromeric TMR-CAL1 intensity per cell by scatter dot plot. Each dot represents an individual cell. Black line: average signal, blue error bars: standard error.

The role of nucleolar CAL1 is not known. One possibility is that newly synthesized CAL1 is sequestered to the nucleolus during interphase and is released upon nucleolar disassembly during prophase in order to mediate CID centromeric assembly, which occurs immediately after, in metaphase. Since depletion of Modulo causes a loss of this nucleolar CAL1 pool, we next investigated whether newly synthesized CAL1 is recruited normally to centromeres. We carried out Modulo RNAi in S2 cells expressing SNAP-CAL1, a tag that allows the distinction between pre-existing and newly synthesized protein pools [12], [22]. Four days after incubation of the cells with Modulo dsRNA or no RNA, the existing SNAP-CAL1 pool was quenched with BTP block. Cells were then chased for 24 h to allow synthesis of new SNAP-CAL1 protein and newly synthesized SNAP-CAL1 was labeled with the fluorescent reagent TMR-star (Fig. 4A). After imaging, the average centromeric TMR-star signal of SNAP-CAL1 was quantified for individual control and Modulo RNAi cells (Fig. 4B). Modulo RNAi caused a 40% decrease in TMR-star CAL1 when compared to control (no RNAi) SNAP-CAL1 cells (p<0.0001, unpaired t-test; n = 102 cells for each condition), leading us to conclude that Modulo contributes to normal centromeric recruitment of newly synthesized CAL1.

### Modulo knock-down by RNAi causes a decrease in centromeric CID

**Figure 5:**
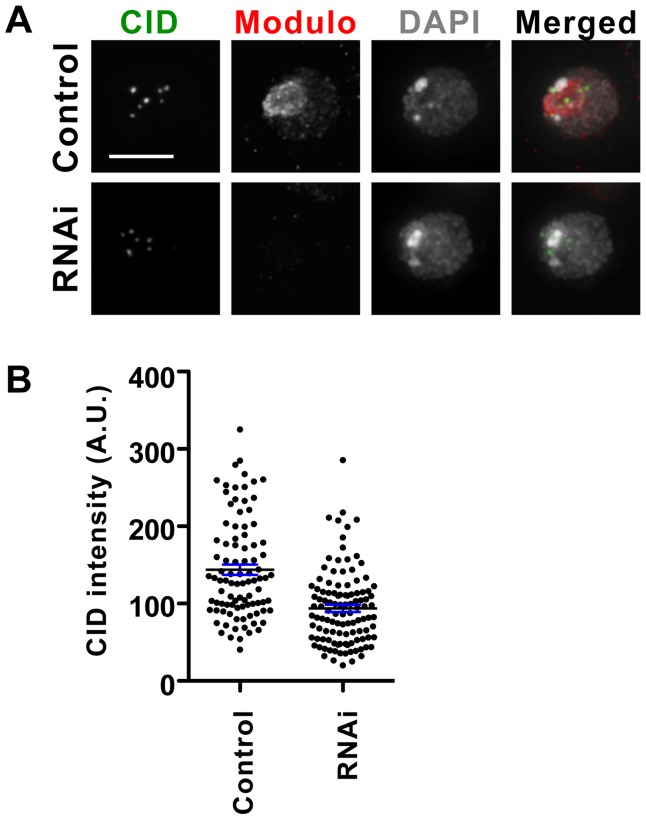
Modulo RNAi causes partial loss of CID from centromeres. A) Modulo was depleted in S2 cells resulting in a decrease in CID signal (green) at centromere as assayed by IF. Staining with anti-Modulo (red) confirmed Modulo depletion in RNAi cells. DAPI is shown in gray. Bar 10 µm. B) Scatter dot plot of the CID signal intensity. Each dot represents the average centromeric CID intensity per cell. Black line: average signal, blue error bars: standard error.

We previously showed that the centromeric localization of CAL1 and CID is inter-dependent [10]. Given that Modulo is required for the proper recruitment of newly synthesized CAL1, we next investigated whether Modulo is important for the normal localization of CID. RNAi of Modulo was performed in S2 cells and centromeric CID was detected by IF. Quantification of the centromeric signal in control cells (n = 93) and Modulo RNAi cells (n = 112) revealed that cells lacking Modulo displayed a 35% decrease in CID signal intensity (p<0.0001, unpaired t-test; Fig. 5A–B), indicating that Modulo contributes to normal CID centromeric levels. Since Western blot analysis showed unchanged total CID protein levels upon Modulo RNAi (Fig. 3A), the observed decrease in CID signal at the centromere could be caused by defective recruitment of newly synthesized CID (referred to as CID assembly) or by defective retention of pre-existing CID at centromeres (referred to as CID maintenance). We tested by RNAi of Modulo RNAi followed by quench-chase-pulse in SNAP-CID expressing cells. Quantification of the TMR-labeled SNAP-CID did not reveal any obvious defect in SNAP-CID assembly at centromeres (p = 0.78, unpaired t-test; Fig. S1). Thus, either the diminished CID intensity observed by IF upon Modulo RNAi is due to defective CID maintenance or the quench-chase-pulse with SNAP-CID is not sensitive enough to reveal a partial defect in CID assembly.

### Chromosome segregation defects in cells lacking Modulo

**Figure 6:**
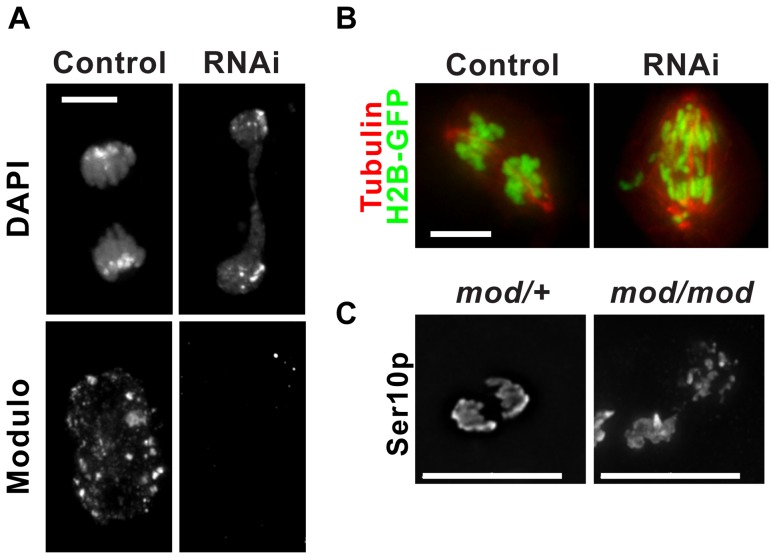
Modulo is required for proper chromosome segregation. A) Modulo was depleted in S2 cells by RNAi and chromosome segregation in mitosis was monitored by IF. Staining with Modulo antibody confirmed the successful depletion. Anaphases in cells lacking Modulo (RNAi) displayed lagging and stretched chromosomes at higher frequency than control cells. Bar 5 µm. B) Chromosome segregation was monitored by timelapse microscopy in S2 cells expressing H2B-GFP and mCherry-tubulin. Representative frames for a control and RNAi video are shown. Bar 5 µm. C) Representative anaphases identifiable by H3 Ser10p staining from brain whole-mounts from third instar larvae of *mod^lethal8^* heterozygote mutants (*mod/+*) and in *mod^lethal8^* homozygote mutants (*mod/mod*). Frequent stretched and lagging chromosomes were observed in the homozygote mutant (see text for details). Bar 15 µm.

Our data so far point to a role of Modulo in regulating proper CAL1 and CID localization. One indication of such a role would be the presence of chromosome segregation defects upon Modulo depletion. To test this, we analyzed chromosome segregation in mitotic cells identified through IF with anti-histone H3 Ser10p antibodies as a mitotic marker. We compared anaphase figures from control (n = 41) and Modulo RNAi cells (n = 51) and observed defective anaphases in 86% of Modulo RNAi cells compared to 49% of control cells (p = 0.0002, Fisher's exact test; Fig. 6A). The type of defects observed in control cells were similar to those in the RNAi, namely lagging and stretched chromosomes, with differences in the frequency and in the severity of the defects, which were higher in the Modulo RNAi. We also determined the rate of chromosome segregation defects by time-lapse microscopy in S2 cells expressing histone H2B-GFP and mCherry-tubulin. In these experiments, control cells displayed no defects (n = 16), while Modulo RNAi-treated cells showed defects in 50% of cells (n = 12; p = 0.0025, Fisher's exact test; Fig. 6B). The main types of defects observed were misaligned chromosomes in metaphase and lagging and stretched chromosomes in anaphase.

To assess the effect of Modulo deletion on chromosome segregation in animals, we used a *modulo* null fly line (*mod^lethal8^*; [40]). Because the *mod^lethal8^* mutation also affects the *krz* gene adjacent to the *modulo* gene, we used a line homozygous for a genomic rescue fragment containing the *krz* gene [41]. Western blot from total larvae extracts and IF on larval brain whole-mounts with anti-Modulo antibodies confirmed the absence of Modulo in homozygotes and its presence in heterozygotes and wild type animals (Fig. S2A–B). Mitotic cells (identified by IF with anti histone H3 Ser10p antibodies) from brain whole-mounts from *mod^lethal8^* homozygous and heterozygous larvae were compared. A higher incidence of chromosome segregation defects was observed in homozygote mutants compared to heterozygotes (*mod^lethal8^*/+; 82%, n = 40 anaphases versus 8.5%, n = 47 anaphases; n = 5 larvae for each genotype, p<0.0001 Fisher's exact test; Fig. 6C), confirming our observations in S2 cells. At lower magnification (20×), the IF CID signal in brain whole-mounts appeared higher in wild type brains compared to *modulo* null brains (Figure S2B), consistent with our observations in S2 cells (Fig. 5). We were not able to assess the localization of CAL1 in *modulo* null flies by IF because of the lack of anti-CAL1 antibodies that work for this technique [10].

We also analyzed the localization of the nucleolar protein fibrillarin in *mod^lethal8^* homozygous larval brains to determine if the lack of Modulo causes gross nucleolar disruption. The localization of Fibrillarin in brain whole-mounts from wild type *modulo* null larvae was unchanged (Fig. S2C), suggesting that the nucleolus is intact when Modulo is absent. These observations lead us to conclude that the mislocalization of nucleolar GFP-CAL1 in Modulo RNAi (Fig. 3) is not due nucleolar disruption, but rather to a lack of the nucleolar partner of CAL1, Modulo.

### Overexpression of Modulo causes severe chromosome segregation defects

**Figure 7:**
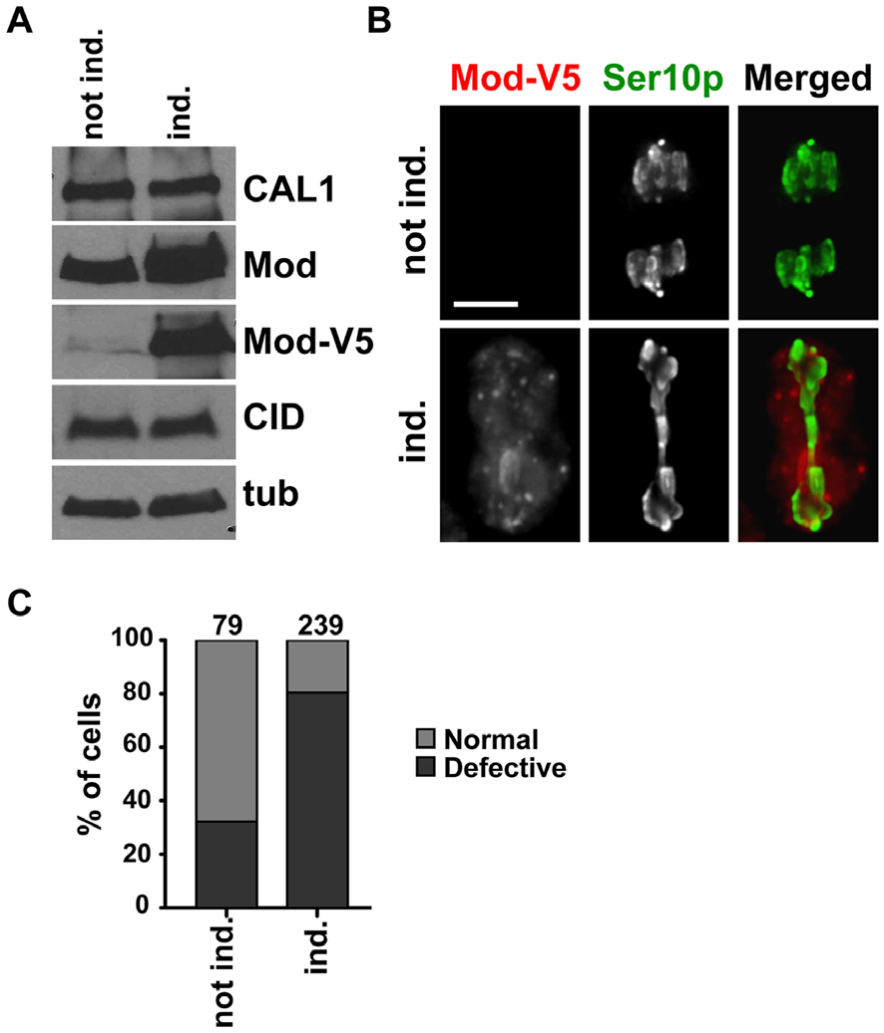
Modulo overexpression causes chromosome segregation defects. A) Western blot to detect Modulo expression in induced (ind.) and uninduced (not ind.) cells stably transfected with pMT-Mod-V5. While CAL1 and CID total protein remained unchanged, Modulo levels increase upon induction when detected with Modulo antibodies. Anti-V5 antibodies show that Modulo-V5 (Mod-V5) is present at very low levels in the unindiced cells and is overexpressed in the induced ones. Western blot with anti-tubulin is shown as a loading control. B) Anaphase defects were observed at higher frequency in cells overexpressing Mod-V5. Representative anaphases from induced (ind.) and uninduced (not ind.) cells where Mod-V5 was visualized by IF with anti-V5 (red) and anti H3 Ser10p (green) identified mitotic cells. Bar 5 µm. C) Percentage of chromosome missegregation in induced (ind.) versus uninduced (not ind.) cells. The numbers above the graph represent the number of anaphases scored for each condition.

Overexpression of proteins can often reveal phenotypes that can help the analysis of a protein's function. We analyzed the effect of Modulo overexpression on centromere integrity and chromosome segregation in S2 cells. The full-length Modulo coding sequence was cloned under the control of a copper sulphate (CuSO_4_) inducible promoter (pMT-V5 vector) and stably-transfected S2 cells harboring the pMT-Modulo-V5 vector were generated. To detect successful Modulo overexpression, cells were either grown in the presence of 500 µM CuSO_4_ overnight or left untreated in growth medium without CuSO_4_ (referred to as uninduced cells) and total extracts were analyzed by Western blot with anti-Modulo and anti-V5 antibodies, confirming the increased levels of Modulo upon induction of the pMT promoter. The Western blot analysis also showed that the overexpression of Modulo does not affect the total levels of CAL1 and CID protein (Fig. 7A). To analyze the effect of Modulo overexpression, stable pMT-Modulo-V5 cells were induced overnight and were then processed for IF with anti-V5, anti-CID and anti H3 Ser10p antibodies. Induced cells showed a strong signal in Modulo-V5 IF, consistent with our Western blots (Fig. 7B). Anaphases were identified among anti H3 Ser10p positive cells and scored manually for the presence of defective chromosome segregation (Fig. 7B). 80% of anaphases from cells overexpressing Modulo displayed dramatic chromosome segregation defects, compared to 32% of uninduced cells (n = 239 and n = 72 respectively; p<0.0001, Fisher's exact test; Fig. 7C). IF with anti-CID antibodies, in induced and uninduced pMT-Modulo-V5 cells, showed no changes in the levels of centromeric CID signal (unpaired t-test p = 0.07; Fig. S3A). Similarly, co-transfection of the GFP-CAL1 and the pMT-Modulo-V5 constructs followed by induction did not cause any obvious changes in GFP-CAL1 localization (unpaired t-test p = 0.15; Fig. S3B). The number of centromeric CID foci was also assessed in induced and uninduced pMT-Modulo-V5 expressing cells. Uninduced and induced cells displayed a virtually identical number of CID foci (4.3±1.4 and 4.2±1.4, respectively n = 102). Collectively, these observations indicate that overexpression of Modulo does not disrupt either centromere structure or centromere clustering and that the observed chromosome missegregation phenotype is likely due to a distinct and unknown function of Modulo.

## Discussion

We report the first study linking the nucleolar protein Modulo to centromere integrity and accurate chromosome segregation. The functional relationship between the nucleolus and the centromere is one of the most poorly understood aspects of centromere biology. In human cells, RNAs produced from alpha-satellite transcription accumulate at nucleoli and are required for the nucleolar enrichment of the centromeric proteins CENP-C and INCENP [17]. The CENP-A assembly factor HJURP also accumulates at the nucleolus during interphase [20], [21]. Centromeres of human cells have a preferential localization near or around nucleoli. This spatial relationship appears to be conserved across species as demonstrated by the fact that centromeres cluster around nucleoli in *Drosophila* cells as well. The essential *Drosophila* centromere factor, CAL1, localizes to both centromeres and the nucleolus [10]. Despite the lack of common ancestry between CAL1 and HJURP, CAL1 has functions and dynamics that are remarkably similar to those of HJURP, which has led to the proposal that CAL1 may fulfill functions analogous to those performed by HJURP [12], [42]. Similarly to CAL1, the functional significance of HJURP's nucleolar localization is unknown. Here, we report the isolation of Modulo, the *Drosophila* nucleolin homolog, from CAL1-bound complexes extracted from chromatin-free extracts. Immunoprecipitations using total nuclear extracts confirmed that Modulo and CAL1 physically interact. Given the relatively low recovery of CAL1 from Modulo immunoprecipitates, these experiments also suggest that only a small proportion of Modulo interacts with CAL1, which is consistent with the observation that Modulo is not only nucleolar-associated, but it is also broadly distributed on chromosomes. Despite the fact that CAL1 and Modulo interact, detailed cytological analyses of cells at different cell cycle stages provided no evidence that Modulo co-localizes with centromeres, suggesting that the interaction between Modulo and CAL1 is restricted to the nucleolar compartment.

The functional significance of the presence of CAL1 at the nucleolus is not clear. The middle region of CAL1 (residues 408–698), which is required for its nucleolar localization [13], is the least conserved among CAL1 orthologs from different *Drosophila* species, when compared to the N (residues 1–407) and C (residues 699–979) termini [12], [13], [42]. When this nucleolar-targeting region is deleted from CAL1, in a construct consisting of the N and C termini fused together, CAL1 still localized to the centromere in S2 cells. Furthermore, the truncated CAL1 transgene lacking the middle region was reported to rescue a fly CAL1 null mutant [13], although it was not reported whether the viability and fertility of the offspring was comparable to those of wild type flies. Nonetheless, these observations suggest that the nucleolar localization of CAL1 is not essential to CAL1's function and that nucleolar and centromeric localization are separable functions within CAL1.

Depletion of Modulo causes a loss of nucleolar CAL1. Since we detected no change in centromeric GFP-CAL1 upon Modulo RNAi and since Western blot analyses showed no change in total CAL1 levels after Modulo RNAi, it is likely that the CAL1 lost from the nucleolus becomes broadly distributed within the nucleus rather than accumulate at the centromere. Regardless of the fate of the mislocalized CAL1, tracking of newly synthesized CAL1 after Modulo depletion by RNAi showed that the recruitment of CAL1 was impaired. Interestingly, CAL1 is replenished at centromeres during prophase [12], the time when the nucleoli disassemble. Thus, it is possible that new CAL1 is stored in the nucleolus and that nucleolar disassembly allows the release of free CAL1 to mediate CID assembly onto the centromere. Consistent with this, Modulo depletion and, consequently, nucleolar CAL1 loss was shown to have a negative effect on centromeric CID levels. It is possible that the cycle of CAL1 in and out of the nucleolus is an important part of its regulation and that Modulo mediates such cycle. CAL1 could for instance become modified while within the nucleolus, a modification that could regulate its centromeric role. CAL1 has indeed been shown to be phosphorylated [43].

CID centromeric levels decrease approximately 35% when Modulo is depleted (Fig. 4). As observed for canonical histones and human CENP-A [22], CID is diluted of 50% at each cell division [12] and therefore, complete failure to recruit CID should result in a 50% decrease in CID signal, which is not what we observed. Thus, either lack of Modulo only partially impairs CID recruitment or the lack of Modulo impairs the ability of centromeres to retain pre-existing CID. Using the SNAP-tag system to track the recruitment of newly synthesized SNAP-CID, we did not detect defective assembly. However, this method might not be sensitive enough to detect subtle defects and thus we cannot entirely rule out that CID recruitment is defective. Collectively our experiments suggest that the absence of Modulo negatively affects the normal CAL1 function of supporting CID chromatin integrity and, perhaps, recruitment of newly synthesized CID. The fact that CID chromatin is not completely lost in the absence of Modulo suggests that despite the mislocalization, CAL1 remains available to at least partially fulfill its function.

Cells lacking CENP-A fail to assemble functional kinetochores and to segregate their chromosomes [44]. Depletion of Modulo in *Drosophila* cells caused a drastic increase in the rate of chromosome segregation defects. The observed rate of chromosome missegregation may be due to the lower levels of CID at centromeres, to additional functions for Modulo in chromosome structure or function, or a combination of these possibilities.

Modulo overexpression caused accumulation of Modulo throughout the nucleus, however quantification of CID and GFP-CAL1 by IF showed no significant changes in signal intensity. These observations suggest that, although Modulo depletion negatively impacts CID localization at the centromere, overexpression does not have the opposite effect of stimulating more CID recruitment. These conclusions are compatible with a model whereby the effect of Modulo on CID levels is due to its role in nucleolar CAL1 retention.

The requirement of Modulo for the nucleolar localization of CAL1 appears to be unique to this nuclear structure as demonstrated by the fact that overexpressed Modulo, which causes additional Modulo protein to localize throughout the nucleus, does not cause mislocalization of CAL1 throughout the nucleus (data not shown). The localization dependency is also one-way, since we observed that Modulo localizes normally within the nucleolus in the absence of CAL1 (data not shown).

Modulo null flies die during pupation [38] whereas CID and CAL1 null mutants die during early embryogenesis [10], [45], reflecting the importance of these two gene products in early development. These phenotypic differences suggest that either the CID and CAL1 maternal products become depleted in null animals earlier than Modulo causing the earlier lethality, or that Modulo's gene function is not essential in the early stages of embryogenesis unlike that of CAL1 and CID. These considerations support a model where animals can cope with defective centromeres at least until pupation despite the lack of Modulo and suggest that partially impaired centromere composition and high rate of chromosome missegregation can be tolerated during the earlier stages of development. Given that Modulo is involved in other processes beyond centromere regulation, we propose that the lethality of Modulo null mutants is a result of defective chromosome transmission and other chromatin and nucleolar dysfunctions.

## Methods

### Large-scale immunoprecipitation and mass spectrometry

FLAG-CAL1 complexes were isolated and purified from chromatin-free extracts generated from 1×10^9^ S2 cells, as described in [12] (protocol available upon request). The eluted FLAG-CAL1 complex was reduced, carboxamidomethylated and digested with trypsin and LysC. Analysis of the digested complex was carried out by LC-MS/MS on a Waters/Micromass AB QSTAR Elite mass spectrometer (Keck Biotechnology Resource, Yale School of Medicine). MS/MS spectra were analyzed using the MASCOT algorithm to search the NCBInr (Drosophila) database for identification of the peptides present in the complex.

### Modulo Immunoprecipitation

Nuclei from 1×10^8^ S2 cells were prepared by pelleting and resuspending cells in 1.5 ml of Buffer A (20 mM Hepes, pH 7.4, 10 mM KCl, 1.5 mM MgCl2, 0.34 M Sucrose, 0.2% Triton X-100, 10% Glycerol,1 mM PMSF, 1× EDTA-free protease inhibitors (Roche) and 1 mM DTT). Cells were homogenized in a dounce homogenizer using 25 strokes and nuclei were pelleted at 600×g. Nuclei were then washed once in dounce buffer (Buffer A containing 150 mM KCl). Nuclei were resuspended in Re-suspension Buffer (0.29 M Sucrose, 0.5 mM Tris-HCl pH 7.4, 1.5 mM NaCl, 5 mM MgCl2, 1 mM EGTA, 0.04% Triton-X-100, 1× EDTA-free protease inhibitors, 1 mM DTT), nuclei were spun at 500×g for 15 min at 4°C followed by resuspension in Solution A (10 mM HEPES, 2 mM MgCl2, 0.25 M Sucrose). A sucrose cushion (10 mM HEPES, 2 mM MgCl2, 0.5 M Sucrose) was placed beneath the nuclei re-suspended in Solution A and nuclei were pelleted at 500×g for 15 min at 4°C. Nuclei were resuspended in salt-free Buffer B (0.2 mM EGTA, 1 mM DDT, 1× EDTA-free protease inhibitors) and incubated for 30 minutes on ice to extract the nucleoplasm. The pellet, which contained the chromatin fraction, was then digested with 4 µl of benzonase (Novagen) in digestion buffer (1× EDTA-free protease inhibitors, 10 mM Tris-HCl, pH 7.4, 0.3 M NaCl, 1 mM MgCl2, 0.025% NP-40) at 4°C for 60 min with gentle rotation. Following benzonase treatment, extracts were supplemented with 2 mM EDTA and centrifuged at 12,000×g for 10 min at 4°C. The supernatant (chromatin extract) was then used as the input in the immunoprecipitations. 350 µg of anti-modulo antibody (gift of Jacques Pradel) was coupled to 1.5 mg of Dynabead Protein-A (Invitrogen) beads (50% slurry), following the manufacturer's instructions. The chromatin extract was incubated with antibody bound beads for 2 h at 4°C. Bound complexes were washed three times with 200 µl cold PBS. 20 µl of Laemmli buffer (without reducing agents DTT or β-mercaptoethanol) was added to the beads and then boiled 5 min at 95°C. 1.3% of the total input and 56% of the total IP were used for analysis by Western blot. Modulo antibodies (1∶1000) and CAL1 affinity purified rabbit polyclonal antibodies ([10]; 1∶1000) were used for detection. Image J was used to quantify the enrichment of Modulo protein in the IP compared to the mock.

### FLAG-CAL1 Immunoprecipitations

1×10^8^ S2 cells stably expressing FLAG-tagged CAL1 (where CAL1 is expressed as a N-terminal fusion with FLAG under the pCopia promoter) and 1×10^8^ S2 cells were washed in PBS. Chromatin extracts were obtained as above and were incubated with anti-FLAG M2 agarose (Sigma) for 2 h at 4°C. Bound complexes were washed and beads were boiled in 20 µL of Laemmli buffer as above. 25% of the total input and 25% of the total IP were used for analysis by Western blot. Modulo antibodies (1∶1000) and anti-FLAG antibodies, Sigma(1∶1000) were used for detection. Image J was used to quantify the enrichment of Modulo protein in the FLAG-CAL1 IPs compared to the IPs from untagged S2 cells.

### Modulo RNAi

dsRNA was prepared using the kit MegaSCRIPT T7 (Ambion) according to the manufacturer's instructions. Templates were generated by PCR from modulo cDNA (DGRC) using the following primers: T7-*modulo* forward-TAATACGACTCACTATAGGGCTCAAGGACGATGAGGGTTTC, T7-*modulo* reverse-TAATACGACTCACTATAGGGCGCTGTGGCCGTATTTATGGT. 2×10^6^ logarithmically growing S2 cells (untransfected or stable transfected lines) were plated in 1 ml of serum-free medium, and 15 µg of dsRNA was added to the culture. Stable lines expressing pCopia-GFP-CAL1 and mCherry-tubulin [10] were used for the experiments in Figure 3. Control wells received water instead of dsRNA. After 30 min of incubation, 1 ml of serum-containing medium was added, and incubation continued for 4 or 5 days. Samples were subjected to indirect IF analysis or live cell imaging (for GFP-CAL1 mCherry-tubulin cells) and Western blot. All experiments were repeated at least twice.

### Western blotting of cell extracts

1×10^6^ cells were resuspended in 15 ul RIPA buffer(150 mM NaCl, 50 mM Tris, pH 8, 1% NP40, 0.1% SDS) and kept on ice for 10 mins. The cell lysates were digested with 1 µl of benzonase (Novagen) for 20 min at 37°C. Extracts were separated by 10% SDS-PAGE and transferred to nitrocellulose membranes. After 30 min block in TBS-T 5% Milk (TBS, 0.1% Tween 20, 5% powder non-fat milk), membranes were incubated overnight at 4°C with anti-Modulo antibodies (mouse, 1∶1000), anti-CAL1 (rabbit, 1∶1000), and anti-CID (rabbit, 1∶1000). Anti-Lamin (mouse, 1∶1000 Hybridoma Bank, Univ. of Iowa) or anti-tubulin (1∶1000, Mouse; Sigma) were used as a loading control.

### Immunofluorescence and Imaging

Cells were pelleted, resuspended in 1× PBS, settled on a glass slide, and fixed with 3.7% formaldehyde in PBS-T (PBS with 0.1% Triton X-100) for 10 min. Slides were washed three times for 5 min in PBS-T, rocking, and then were blocked in 5% milk in PBS-T for 20 min. Slides were incubated with 30 µl of PBS-T 5% milk containing the appropriately diluted primary antibodies. Slides were washed three times for 5 min in PBS-T, with gentle rocking, and then were incubated with secondary antibodies (all Alexa conjugated antibodies from Molecular Probes, 1∶500 dilution) for 45 min at room temperature in a humid chamber. Slides were washed three times for 5 min in PBS-T, with gentle rocking, and were then mounted on coverslips with SlowFade Gold Reagent (Invitrogen) containing 2.9 µM DAPI.

Chromosome spreads were obtained using 2×10^5^ cells per slide resuspended in 475 µl of serum medium and incubated with 25 µl of Colcemid (20×-Sigma) for 1 h. After Colcemid treatment, cells were spun for 5 min at 600×g at room temperature and resuspended in 250 µl of 0.5% Sodium Citrate for 8 min. After incubation, cells were placed in a cytofunnel and spun at 1200 rpm for 5 min using a Shandon Cytospin 4 (Thermo Scientific). Cells were immediately fixed in 3.7% formaldehyde and processed for IF as above.

Antibodies used were: anti-Modulo (Mouse, 1∶150, gift of Jacques Pradel or chicken, 1∶100, gift of Dmitry Nurminski [36]), anti-CID (Chicken, 1∶500, [2]), anti H3 Ser10p (Rabbit, 1∶1000; Millipore # 06-570), anti GFP-488 conjugated (Invitrogen, 1∶500), anti-Fibrillarin (Mouse, 1∶500, Cytoskeleton, Inc.).

Slides were imaged using a 60×/1.42 or a 100×/1.40 oil immersion Olympus objective on a PersonalDV microscope (Applied Precision) keeping exposure constant between all samples. Images were scaled in Softworks, maintaining the scaling constant between samples, saved as. psd files and figures were assembled in Adobe Illustrator.

Time-lapse videos were performed on a PersonalDV microscope using a 60×/1.42 objective. mCherry-tubulin and H2B-GFP expressing cells (gift of Gotha Goshima) subjected to Modulo RNAi were mounted using the hanging drop method [46]. Cells were imaged every 1 or 2 min until cytokinesis for a total of 30–45 min.

### Detection of newly synthesized SNAP-CAL1 and SNAP-CID (quench-chase-pulse)

*Drosophila* Schneider (S2) cells [47] stably expressing SNAP-tagged CAL1 or SNAP-CID were treated with 15 µg of dsRNA against Modulo using the ‘soaking method’ described above. The quench-chase-pulse experiments were carried out essentially as described previously using the SNAP-tag kit by NEB [12]. Briefly, SNAP-proteins were quenched with BTP after 4 days of RNAi depletion. BTP was washed away, and cells were allowed to grow for another 24 h to allow synthesis of new SNAP-protein, which was then detected by TMR* labeling. Cells were fixed with 3.7% formaldehyde in PBST for 10 min, and Modulo was detected by IF used at 1∶1000.

### Quantification of centromeric and nucleolar signal for GFP-CAL1

Images were deconvolved using Softworx (Applied Precision) selecting the ‘conservative’ mode, with 5 iterations and quick projected selecting the maximum intensity setting. Using the 2D Model function, polygons were generated for individual cells in the DAPI channel to encompass the entire DAPI area. The polygons were then propagated in the green channel (for GFP-CAL1) using the polygon editor tool. The GFP intensity per cell was calculated as follows: from the total GFP nuclear intensity the diffuse nucleoplasmic signal (minimum intensity value×DAPI area) was subtracted. The resulting value represents the sum of nucleolar and centromeric signal for GFP-CAL1 (total GFP-CAL1). The centromeric signal of GFP-CAL1 was quantified by generating centromeric polygons for individual cells in the GFP channel and by adding the total GFP intensity per centromeres together for each cell. The nucleolar GFP intensity was then calculated by subtracting the centromeric GFP intensity from the total GFP-CAL1. Intensities (arbitrary units) were plotted and analyzed in Prism (Graph pad).

### Quantification of TMR signal for SNAP-CAL1

Images were deconvolved and quick projected as above. Using the 2D Model function, polygons were generated for individual cells in the TRITC channel to select the centromeric dots. The centromeric TMR value for each cell was calculated by the averaging the two strongest centromeric spots for SNAP-CAL1. For individual cells, the centromeric TMR intensity was calculated by subtracting the background for the TRITC channel (minimum intensity value×polygon area) from the centromeric TRITC intensity.

### Quantification of CID signal after modulo RNAi

In Softworx Suite, images were deconvolved with the method set to conservative ratio, the number of cycles set to 5, and noise filtering set to medium. Images were then quick projected with the method set to max intensity. The control field scale was copied and applied to all fields. The 2D polygon finder (Softworks) was used to process the deconvolved, quick projected images. DAPI masks were created by selecting the DAPI channel, setting the minimum perimeter set to 100 (12.92 µM), excluding outer edge objects, and applying an appropriate threshold. The DAPI polygons were propagated through the H3 Ser10p channel for exclusion of mitotic cells from the quantification. To ensure only cells exhibiting the Modulo RNAi phenotype were included in the quantification, DAPI polygons were propagated into the Modulo channel, and Modulo positive cells were excluded. The resulting Modulo-lacking, interphase, DAPI mask polygons were then propagated through the CID channel. DAPI masks were cut to include the CID wavelength. The control field scale was applied and the images were exported as TIFF files with scaling using min/max/exp values, the destination computer set as Mac/SGI, and the output size set as 8-bit grey. The TIFF files were analyzed using the Image Analysis Tool (from Colin Fuller and Aaron Straight) to measure the CID intensity per centromere. Values were exported as a text file and imported into Microsoft Excel. The background intensity value was subtracted from the quantified channel value for the CID intensity per cell. The CID intensity per cell was divided by the number of centromeres per cell which resulted in the CID intensity per centromere.

### Quantification of CID signal after Modulo overexpression

Images were deconvolved using Softworx (Applied Precision) selecting the ‘conservative’ mode, with 5 iterations and quick projected selecting the maximum intensity setting. Using the 2D Model function, polygons were generated for individual cells in the DAPI channel to encompass the entire DAPI area. The polygons were then propagated through the CID channel using the polygon editor tool. Values were imported into Microsoft Excel. The background intensity value was subtracted from the quantified channel value for the CID intensity per cell.

### Modulo null mutants IF and total extracts

*mod^lethal8^* mutants harboring a genomic fragment containing wild type krz P(krz:B5,8T12); *mod^lethal8^*/SM5-TM6B were grown and tubby (*mod^lethal8^*/SM5-TM6B heterozygotes) and non-tubby (*mod^lethal8^*/*mod^lethal8^* homozygotes) third instar larvae were dissected in PBS and whole brains were processed for IF with anti-Modulo and anti H3 Ser10p antibodies as described in [46] or were homogenized with a pestle, resuspended in Laemmli buffer, boiled at 95°C for 5 minutes and processed for SDS-PAGE and Western blot. The 1118 fly line was used as a wild type (+/+) control.

## Supporting Information

Figure S1 **Newly synthesized SNAP-CID is recruited normally at centromeres upon Modulo RNAi.** RNAi of Modulo was performed in cells expressing SNAP-CID. Newly synthesized CID was tracked by TMR labeling following a quench and chase of SNAP-CID protein. Quantification of the TMR-CID signal (shown by scatter dot plot) shows no detectable defect in SNAP-CID recruitment. Black line: average signal, blue error bars: standard error.
(TIF)

Figure S2 **Analysis of Modulo, CID and Fibrillarin localization in** ***mod^lethal8^*** **/** ***mod^lethal8^*** **null larvae.** A) Total larvae protein extracts were generated from wild type (1118), *mod^lethal8^*/+ heterozygotes and *mod^lethal8^*/*mod^lethal8^* nulls. Extracts were resolved by SDS-PAGE and Western blot was performed using anti-Modulo antibody. Increasing amounts (as shown) were loaded. Modulo nulls have no visible Modulo protein, while *mod^lethal8^*/+ heterozygotes have less Modulo than wild type larvae. The asterisk indicates the position of a non-specific band. Western blotting with anti-Lamin antibodies is shown as a loading control. B) IF was carried out on whole-mount brains from wild type (*+/+*) and Modulo null mutants (*mod^lethal8^*/*mod^lethal8^*) with anti-Modulo (red), anti-CID (green) antibodies and DAPI (blue). Images show comparable regions of the central ganglion imaged with 20× magnification. Modulo null mutants showed a complete lack of visible Modulo signal, while overall CID staining appears reduced in these animals. Bar 20 µm. C) IF was performed on whole-mount brains from wild type (*+/+)* and *modulo* null mutants (*mod^lethal8^*/*mod^lethal8^*) with anti-Fibrillarin (red), anti-CID (green) and DAPI (blue). Fibrillarin staining appears similar in wild type and Modulo null flies. Bar 5 µm.
(TIF)

Figure S3 **Overexpression of Modulo does not affect CID or GFP-CAL1 intensity.** A) Quantification of the CID signal (shown by scatter dot plot) shows no increase in CID signal upon Modulo-V5 induction (ind.) compared to uninduced cells (not ind.). B) Quantification of the GFP-CAL1 signal shows no increase in GFP-CAL1 signal. Black line: average signal, blue error bars: standard error.
(TIF)
