# Supplementary figures and images for: A Role for the CAL1-Partner Modulo in Centromere Integrity and Accurate Chromosome Segregation in Drosophila
Source: PLoS One. 2012 Sep 21;7(9):e45094. doi: 10.1371/journal.pone.0045094 (PMC3448598; doi:10.1371/journal.pone.0045094)

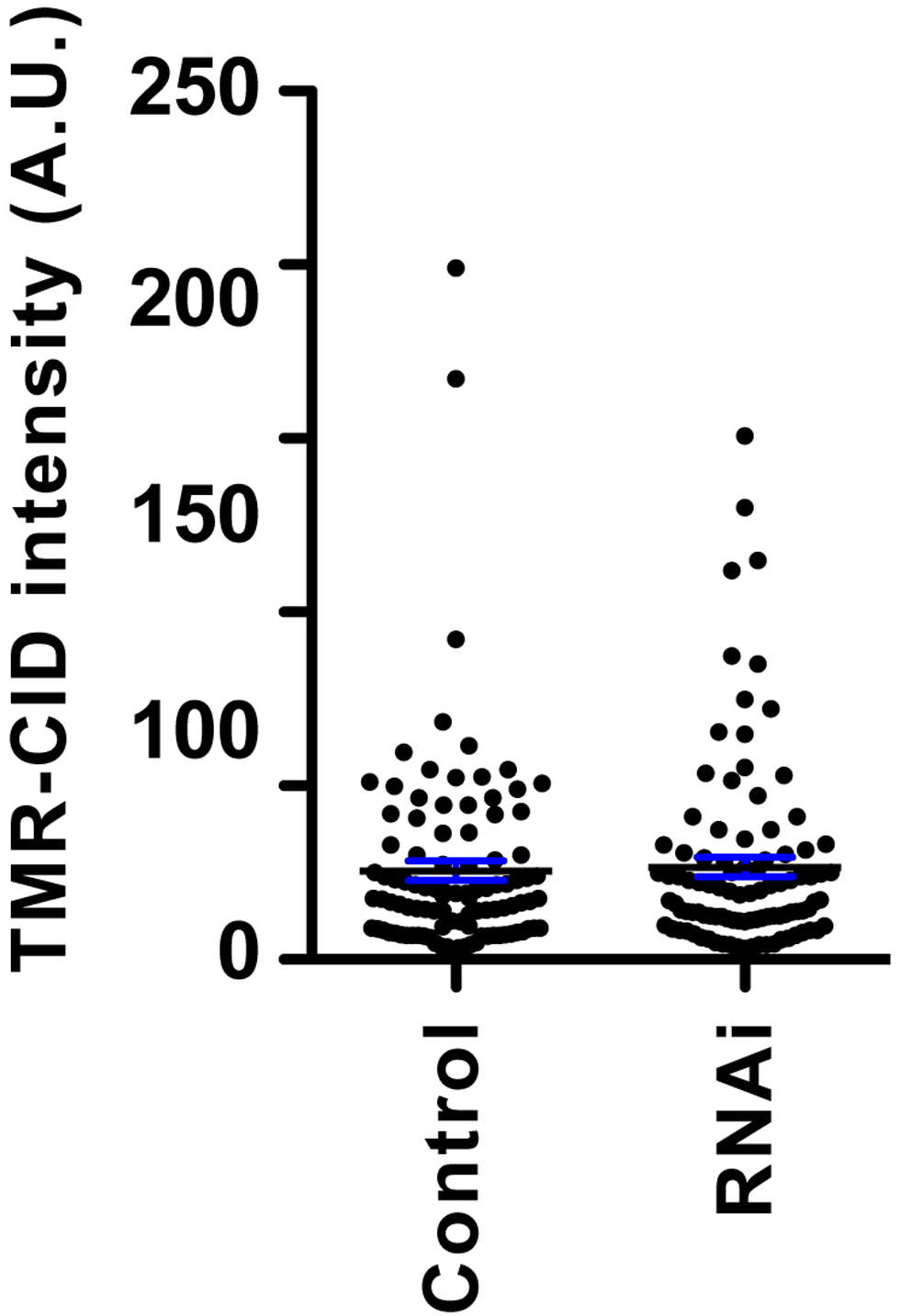

Supplement: Figure S1 — Newly synthesized SNAP-CID is recruited normally at centromeres upon Modulo RNAi. RNAi of Modulo was performed in cells expressing SNAP-CID. Newly synthesized CID was tracked by TMR labeling following a quench and chase of SNAP-CID protein. Quantification of the TMR-CID signal (shown by scatter dot plot) shows no detectable defect in SNAP-CID recruitment. Black line: average signal, blue error bars: standard error. (TIF) [file pone.0045094.s001.tif]

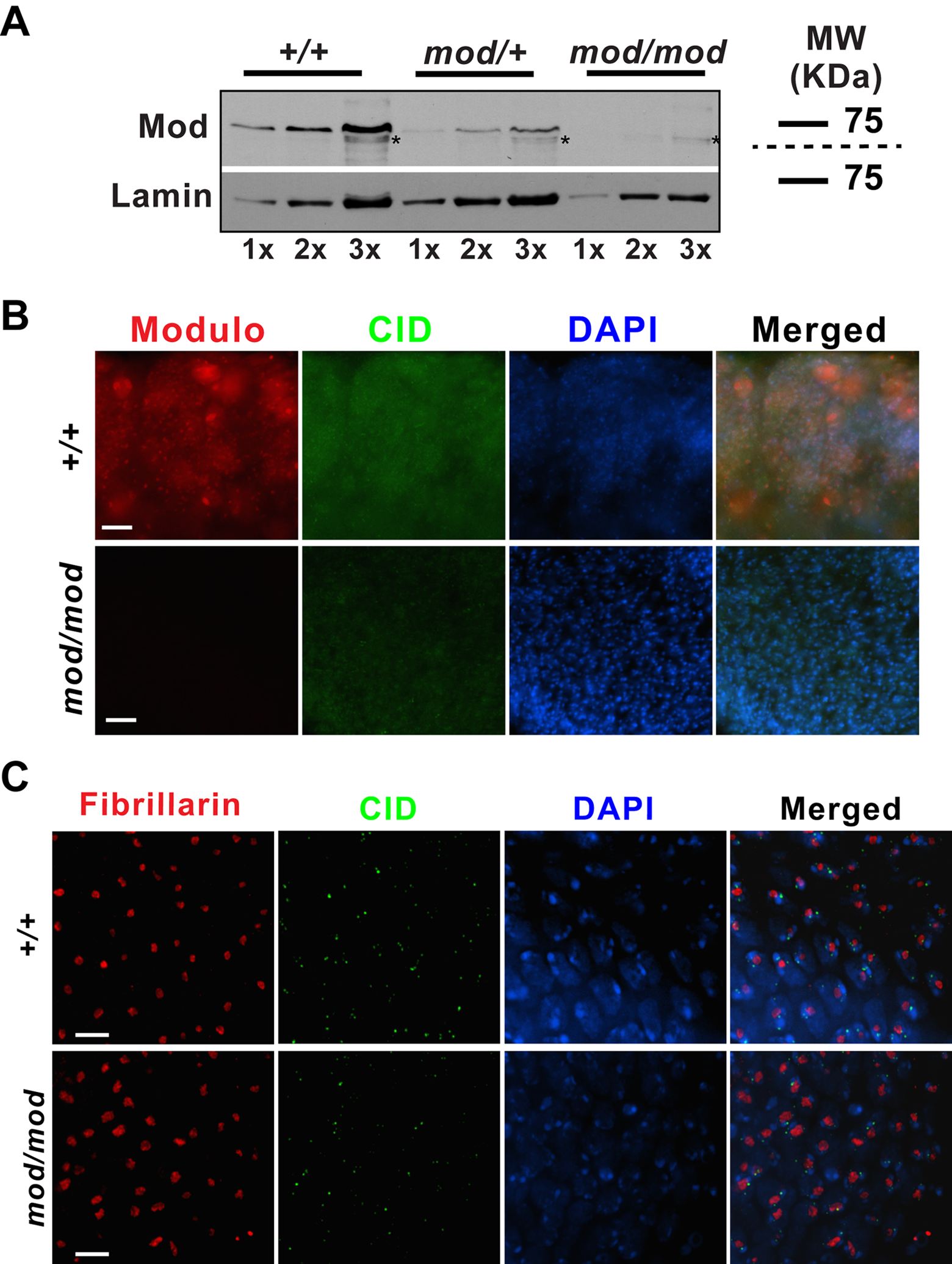

Supplement: Figure S2 — Analysis of Modulo, CID and Fibrillarin localization in modlethal8 / modlethal8 null larvae. A) Total larvae protein extracts were generated from wild type (1118), modlethal8/+ heterozygotes and modlethal8/modlethal8 nulls. Extracts were resolved by SDS-PAGE and Western blot was performed using anti-Modulo antibody. Increasing amounts (as shown) were loaded. Modulo nulls have no visible Modulo protein, while modlethal8/+ heterozygotes have less Modulo than wild type larvae. The asterisk indicates the position of a non-specific band. Western blotting with anti-Lamin antibodies is shown as a loading control. B) IF was carried out on whole-mount brains from wild type (+/+) and Modulo null mutants (modlethal8/modlethal8) with anti-Modulo (red), anti-CID (green) antibodies and DAPI (blue). Images show comparable regions of the central ganglion imaged with 20× magnification. Modulo null mutants showed a complete lack of visible Modulo signal, while overall CID staining appears reduced in these animals. Bar 20 µm. C) IF was performed on whole-mount brains from wild type (+/+) and modulo null mutants (modlethal8/modlethal8) with anti-Fibrillarin (red), anti-CID (green) and DAPI (blue). Fibrillarin staining appears similar in wild type and Modulo null flies. Bar 5 µm. (TIF) [file pone.0045094.s002.tif]

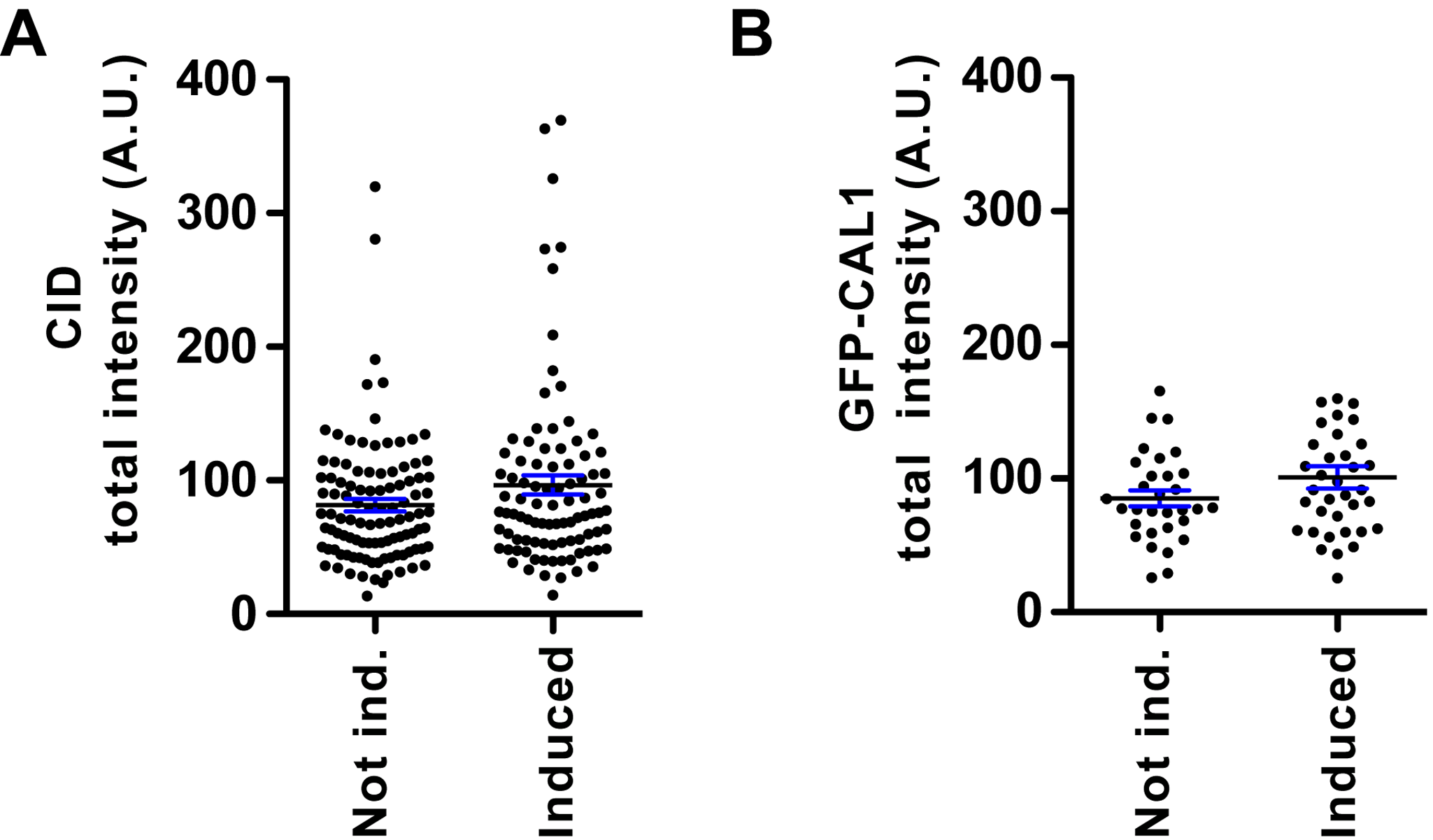

Supplement: Figure S3 — Overexpression of Modulo does not affect CID or GFP-CAL1 intensity. A) Quantification of the CID signal (shown by scatter dot plot) shows no increase in CID signal upon Modulo-V5 induction (ind.) compared to uninduced cells (not ind.). B) Quantification of the GFP-CAL1 signal shows no increase in GFP-CAL1 signal. Black line: average signal, blue error bars: standard error. (TIF) [file pone.0045094.s003.tif]
